# Patients’ preferences and willingness-to-pay for postmenopausal hormone receptor-positive, HER2-negative advanced breast cancer treatments after failure of standard treatments

**DOI:** 10.1186/s40064-015-1482-9

**Published:** 2015-11-05

**Authors:** Surachat Ngorsuraches, Klangjai Thongkeaw

**Affiliations:** Department of Pharmacy Practice, College of Pharmacy, South Dakota State University, Brookings, SD, 57007 US; Maharaj Nakhon Si Thammarat Hospital, Nakhon Si Thammarat, 80000 Thailand

**Keywords:** Breast cancer, Patient preference, Exemestane, Everolimus, mTOR inhibitor

## Abstract

Patients’ preferences increasingly play roles in cancer treatments. The objective of this study is to examine breast cancer patients’ preferences and willingness-to-pay (WTP) for postmenopausal hormone receptor-positive, HER2-negative advanced breast cancer treatments after failure of standard treatments. Four attributes, i.e. progression free survival (PFS), anemia, pneumonitis, and cost, and their levels of exemestane and exemestane plus everolimus from literature and patient interviews were used to develop a discrete choice experiment questionnaire. Each questionnaire was composed of seven choice sets and each choice set contained those four attributes with different levels. Breast cancer patients were asked to choose one treatment alternative in each choice set. Multinomial logit model was used to determine relative preferences of each attribute and the WTP for all attributes and treatments were calculated. A total of 146 patients were included in study analyses. Results showed that the patients preferred treatments with higher PFS and lower side effects. The patients were willing to pay US$151.6, US$69.8, and US$278.3 per month in exchange for every 1 month increase in PFS and every 1 % decreased risk of anemia and pneumonitis, respectively. The patients were willing to pay for exemestane and exemestane plus everolimus US$551.8 and US$414.2 per month, respectively. In conclusion, patients weighted importance on PFS, anemia, and pneumonitis, when they needed to choose an aromatase inhibitor plus mammalian target of rapamycin (mTOR) inhibitor for advanced breast cancer treatments after failure of standard treatments. They valued exemestane alone more than exemestane plus everolimus.

## Background

Breast cancer is the most common cancer in women worldwide. In 2012, globally the incidence rate and mortality rate of breast cancer was 38.9 and 13.0 per 100,000 (Ferlay et al. 2013). In Thailand, while the incidence rate was relatively lower (22.4 per 100,000), the mortality rate was slightly higher (13.8 per 100,000) (Ferlay et al. 2013). The 5-year prevalence was 54,269 cases in the country (Ferlay et al. 2013). Among all breast cancer patients in Thailand, metastatic breast cancer accounted for 8.8 %, which tended to require advance and costly therapy (National Cancer Institute, Thailand 2011).

Immunohistochemistry has advanced breast cancer prognosis and treatments by identifying hormone receptor (HR) oestrogen receptor (ER) and/or progesterone (PR) and human epidermal growth factor receptor HER2 pathways (Park et al. 2012; Dedes et al. 2011). Based on these receptor pathways, breast cancers are classified into four subtypes—luminal A (ER+ and/or PR+, HER2−, Ki-67 < 14 %), luminal B (ER+ and/or PR+, HER2−, Ki-67 ≥ 14 % or ER+ and/or PR+, HR2+), HER2-enriched (ER−, PR−, HER2+), and triple-negative breast cancer (TNBC) (ER−, PR−, HER2−). They accounted for 59.3, 12.3, 13.3, and 15.1 % of Thai women, respectively (Chuthapisith et al. 2012). The use of treatments targeting the ER and HER2 in patients with early-stage breast cancer has resulted in noticeable reductions in tumor recurrence and death, but resistance to these treatments often due to the activation of alternative survival pathways can develop and cause disease progression (Dhillon 2013). These pathways have been a focus for the development of new anticancer treatments.

Among those pathways, the phosphatidylinositol 3-kinases (PI3 K)/Akt/mammalian target of rapamycin (mTOR) pathway plays an important role in breast cancer cell proliferation and cancer treatment resistance (Dhillon 2013). The inhibitor of mTOR was shown to restore breast cancer cells’ sensitivity to hormone. An mTOR inhibitor, everolimus, has become a promising treatment for several types of tumors (Dhillon 2013). Everolimus was first approved only for patients with advanced renal cell carcinoma after failure of treatment with sunitinib or sorafenib or vascular endothelial growth factor (VEGF) targeted therapy in several countries as same as in Thailand (Dhillon 2013; Baselga et al. 2012; Bachelot et al. 2014). In 2012, it was approved in the US for the treatment of postmenopausal women with advanced hormone receptor-positive, HER2-negative breast cancer in combination with exemestane after failure of standard treatments, e.g. letrozole or anastrozole (National Cancer Institute 2013). In 2013, the Breast cancer trials of OraL Everolimus-2 (BOLERO-2) data showed the results from final progression-free survival (PFS) analysis of everolimus used in patients with postmenopausal hormone receptor-positive, HER2-negative advanced breast cancer (Yardley et al. 2013). The addition of everolimus to exemestane significantly prolonged median PFS, as compared to exemestane alone (7.8 vs 3.2 months by local investigators and 11.0 vs 4.1 months by central assessment). After final 18-month follow-up, the percentage of overall survival (OS) events of everolimus plus exemestane was 25.4 % while the OS of exemestane alone was 32.2 %. While the most commonly reported adverse events of the everolimus plus exemestane included stomatitis, rash, fatigue, diarrhea, nausea, decreased appetite, weight loss, and cough, the most common grade 3 and 4 adverse events were stomatitis, fatigue, dyspnea, anemia, hyperglycemia, and gammaglutamyltransferase increase.

While the technology of cancer treatments has been moving forward and available at high costs, countries need to manage healthcare budgets efficiently. Thailand is not different from other countries and it needs even more resources since it also has provided universal health coverage to every citizen. Even though the data of public health expenditure on cancer did not exist, there were two examples showing that the country has focused heavily on cancer treatments and had a difficulty to ensure access to the treatments. First, in 2008, Thailand needed to grant compulsory licenses for four drugs, which also included letrozole and docetaxel for breast cancer. Even though the public positively viewed this policy and its benefits, it was a contentious decision (Chalkidou et al. 2014). Another example was that the country has used health technology assessment (HTA) as a decision making tool for allocating resources efficiently and it had immense influence on cancer preventions, treatments, and consequences in the country (Chalkidou et al. 2014). One of study results, specifically related to breast cancer, showed that once-in-a-lifetime population-based mammographic screening for women aged either 40−49 or 50−59 years did not have a good value in Thailand (Chalkidou et al. 2014). Historically, while Thai government and pharmaceutical industry had debated between treatment benefits and costs of cancer treatments, patients were rarely involved. As taxpayers, patients should be able to share their decisions. More attention should be given to patients’ preferences for treatment options, especially new and costly treatments. Also, if the government decided not to cover those innovative cancer treatments, patients would assume very high cost burden. The objective of this study was therefore to understand how patients’ preferences for characteristics of postmenopausal hormone receptor-positive, HER2-negative advanced breast cancer treatments after failure of treatment with letrozole or anastrozole and to estimate their willingness-to-pay (WTP) by using a discrete choice experiment (DCE). Specifically, exemestane plus everolimus was used as a case study.

## Results

### Patients’ characteristics

A total of 155 breast cancer patients were invited to participate in this study. However, only 146 breast cancer patients were included for data analyses. The rest had wrong answers for the validity testing choice set. Table 1 shows patients’ characteristics. The overall study patients’ average age was 53.2 years old. More than 65 % of them were married. The majority of patients had only primary school degree. Most of them either owned business or worked in agriculture. Their average monthly income was US$355.2. Almost 80 % of them were under the universal health coverage provided by Thai government and more than a half perceived that they had good health status, as compared to others at the same age. In average, they were diagnosed with breast cancer about 3 years. Almost 50 % of them were having hormonal therapy.

**Table 1:** Patients’ characteristics (N = 146)

Variables	
Age (years, mean ± SD)	53.2 ± 10.2
Marital status [N (%)]	
Married	96 (65.8 %)
Single/widowed/divorced/separated	50 (34.2 %)
Educational level [N (%)]	
Primary school	97 (66.4 %)
Secondary school/high school/diploma	40 (27.4 %)
College/university or higher	9 (6.2 %)
Occupation [N (%)]	
Civil servant	3 (2.1 %)
Private firm	32 (21.9 %)
Own business/agriculture	84 (57.5 %)
No occupation	27 (18.5 %)
Monthly income (US$, mean ± SD)	355.2 ± 2253.7
Health insurance [N (%)]	
Universal coverage	113 (77.4 %)
Civil servant medical benefit scheme	13 (8.9 %)
Others	20 (13.7 %)
Perceived current health status [N (%)]	
Poor	4 (2.7 %)
Fair	55 (37.7 %)
Good	77 (52.7 %)
Very good	10 (6.8 %)
Average years of breast cancer diagnosed (years, mean ± SD)	3.0 ± 3.1
Current breast cancer treatment [N (%)]	
Surgery	1 (0.7 %)
Chemotherapy	18 (12.3 %)
Hormonal therapy	68 (46.6 %)
No treatment at the time being	47 (32.2 %)
Do not know	11 (8.2 %)

### Patients’ preferences

The results of the multinomial logit model are presented in Table 2. All patients seemed to understand the choice tasks well. Approximately 42 % of all observations chose neither treatment alternative, while 58 % chose the either first or second alternatives in the choice sets. All estimated coefficients had expected signs and were statistically significant in every model. The positive signs of the PFS parameter indicated that the patients preferred postmenopausal hormone receptor-positive, HER2-negative advanced breast cancer treatments with higher PFS. The negative signs of the anemia, pneumonitis, and cost parameters reflected that they preferred lower side effects and paying less money for the treatments. The coefficient strength of each attribute can be interpreted as its preference weight on patients’ utility when other attributes are assumed constant. According to the coefficient strengths on the same scales of the anemia and pneumonitis risks, patients weighted on pneumonitis risk higher than on anemia risk of treatments, as indicated by the higher coefficient value (−0.29 VS −0.07).

**Table 2:** Estimated parameters of multinomial logit model

Attribute	β-coefficient	Standard error	*p* value
Progression free survival	0.16074	0.01788	0.0000
Anemia	−0.07331	0.03306	0.0266
Pneumonitis	−0.29558	0.05250	0.0000
Cost per month	−0.32169 × 10^−4^	0.16383 × 10^−5^	0.0000

Log-likelihood = −445.21, Akaike information criterion = 1.03, Pseudo-R^2^ = 0.53

Table 3 shows the WTP for individual attributes of postmenopausal hormone receptor-positive, HER2-negative advanced breast cancer treatments after failure of treatment with letrozole or anastrozole. The patients’ preferences for both treatment effectiveness and adverse events were put in WTP space to make easier for comparison. Marginal WTP for each attribute was calculated to reflect how much respondents were willing to pay for a unit change of each attribute. The results showed that patients were willing to pay for $151.6 for every 1 month of PFS. On the other hand, they were willing to pay US$69.8 and US$278.3 for every one person in 100 persons to avoid anemia and pneumonia or pneumonitis risks from getting the treatments. Then, we obtained the attribute levels of both exemestane and everolimus plus exemestane from literature (Baselga et al. 2012; Bachelot et al. 2014; National Cancer Institute 2013; Yardley et al. 2013) to calculate the WTP and found that the patients in this study were WTP for exemestane and exemestane plus everolimus US$551.8 and US$414.2 per month, respectively.

**Table 3:** Patients’ willingness-to-pay per month for one level change of each attribute (US$)

	Progression free survival	Anemia	Pneumonitis
Average willingness-to-pay	151.6	−69.8	−278.3
95 % confidence interval	121.1–182.2	(−131.6)–(−8.2)	(−374.8)–(−183.9)

## Discussions

To our best knowledge, this was one of very few studies to elicit breast cancer patients’ preferences on their treatments using DCE. Recently, there have been emerging breast cancer treatments with some certain risks, especially adverse events. The results of this study could help clinicians choose the treatments, based on patients’ preferences of both clinical outcomes and economic burden.

This study intended to examine patients’ preferences for attributes of postmenopausal hormone receptor-positive, HER2-negative advanced breast cancer treatments after failure of treatment with letrozole or anastrozole and to estimate their WTP for the treatments. Interestingly, when patients learned about the benefits and risks of the treatment options that they had—aromatase inhibitor vs aromatase inhibitor plus mTOR inhibitor—for this type of breast cancer after failure of standard treatments, they tended to agree what attributes were important to them. We could conclude within interviewing only six patients. Among various benefits, patients focused more on PFS than on clinical benefit rate and on overall survival (compared to placebo). These results were consistent with medical literature, which considered PFS as the primary outcome for these treatments. Interestingly, among several kinds of adverse events, patients prioritized anemia and pneumonitis, which were two most common grade 3 or 4 adverse events from aromatase inhibitor plus mTOR inhibitor (Dhillon 2013). A reason could be that when they imagined they already suffered so much from having advanced breast cancer and they probably did not want any additional disease or symptom that could worsen their health. Also, among the adverse events they learned, anemia and pneumonitis would possibly sound more severe than others, e.g. stomatitis, hyperglycemia, etc.

The study results suggested that patients had different preferences for the attributes of postmenopausal hormone receptor-positive, HER2-negative advanced breast cancer treatments after failure of treatment with letrozole or anastrozole. They were willing to trade among these attributes when they needed to choose the treatments. The results were intuitive that patients preferred higher treatment efficacy (PFS), lower adverse events (anemia and pneumonia or pneumonitis), and lower cost. Based on the same unit of measurement in the DCE, apparently patients weighed more on pneumonitis than on anemia. One of the reasons could be that they could have heard about or had experience or knew someone who had pneumonitis in the past, while anemia was generally rare and probably harder for the patients to perceive its consequences. On the other hand, we could not directly compare the importance between PFS and the adverse event due to their different units of measurement. However, from the ratios of their estimated parameters, we found that the maximum levels of anemia and pneumonitis, which patients were willing to accept in order to increase one month of PFS, were approximately 2 and 0.5 %, respectively. In other words, patients could accept higher risk for anemia to trade for PFS. Our results were inconsistent with the results from an only previous DCE study on breast cancer patients’ preference for prophylactic granulocyte colony-stimulating factors (Johnson et al. 2014). Even though they might not be a good comparison due to various differences, e.g. populations, it was worth noting that the patients in that study weighed treatment effects on blood cells (neutropenia) more than on infection while our patients cared treatment effects on inflammation (pneumonitis) than on blood cells (anemia). An explanation could be that patients might have different preferences on treatment and prophylaxis. For prophylaxis, patients might concern more on neutropenia because it could be a major disruption for their main treatment. On the other hand, for treatment, patients might consider that it was their main treatment and they should focus more on the adverse event that they have been familiar with.

The results indicated that while patients would like to have longer PFS, they had more concern on pneumonitis, which they might have from using the exemestane plus everolimus instead of exemestane alone. Even though the patients in this study had relatively low monthly incomes, they were willing to pay about a half of all their average monthly income to trade for a month of PFS. Unfortunately, the small sample size in this study did not allow us to examine the association between income and WTP. Since the patients’ WTP for not having both anemia and pneumonitis were high, these amounts finally made them willing to pay monthly for exemestane plus everolimus lower than for exemestane alone. Interestingly, while the WTP for exemestane alone calculated from this study was almost four times higher than its cost (US$140.2), the WTP for exemestane plus everolimus was extremely lower than its cost (US$4712.1) in Thailand. These costs were obtained and estimated from the (Ministry of Public Health 2013). Recently the estimated monthly costs for exemestane and exemestane plus everolimus from the same source declined to US$102.1 and US$2874.3, respectively. Ones could argue that WTP may not be a good estimation for market price. However, as a simple cost-benefit analysis, the interpretation of the results should be that the patients valued the benefits for exemestane more than its cost, but they valued the benefits for exemestane plus everolimus much less than its cost. In other words, they realized net costs of exemestane plus everolimus, which meant it was not worth their value of money in case they had advanced breast cancer treatments after failure of standard treatments. One of the reasons that the patients might perceive that everolimus cost them more than its additional benefits to exemestane could be that the additional PFS from adding everolimus was only approximately 7 months, which were relatively short, as compared to other additional risks that patients could have during those months. It was worth mentioning here that the majority of these patients were under universal health coverage scheme sponsored by Thai government. It could be a reason that their WTP were low.

This study suffered from various limitations. First, even though DCE used in this study is a state-of-the-art stated preference method, ones may always argue that it does not reveal or reflect true preference or value since all decisions are not really made. However, this study already tried to minimize validity threats as many as possible, e.g. providing an opt-out alternative in each choice set, which resembled real-life choices. Second, all choice sets comprised only limited number of treatment attributes. There were other attributes that could also affect patients’ preferences. Further research should examine more attributes, which might provide better understandings of patients’ preferences and their WTP. However, the patient interviews would reassure that this limitation should be negligible. Third, since it was not possible to include only patients, who had postmenopausal hormone receptor-positive, HER2-negative advanced breast cancer treatments after failure of treatment with letrozole or anastrozole, patients in this study could be at any stage of breast cancer. They might not be good representatives for this specific type and stage of breast cancer. However, as stated in the methods, we described the disease condition to ensure that they could imagine well while they were making decisions. Finally, this study examined preferences among patients from only one hospital in southern Thailand, the results could not be generalized to patients in other settings since they might have different socioeconomic characteristics, especially income and education levels, which could affect patients’ preferences and WTP.

In conclusion, this study revealed that patients weighted importance on PFS, anemia, and pneumonitis, when they needed to choose an aromatase inhibitor plus mTOR inhibitor for advanced breast cancer treatments after failure of standard treatments. Specifically, they valued net benefits for exemestane, but not for exemestane plus everolimus. Also, they were willing to pay more for exemestane alone than for exemestane plus everolimus.

## Patients and methods

DCE has been used to measure the preferences of respondents for various treatment alternatives from well-designed choice sets in the healthcare field. This study followed a user’s guide for DCE published by Lancsar and Louviere (Lancsar and Louviere 2008). In general, DCE describes various choice sets of a treatment by its attributes, e.g. efficacy, side effects, and costs. Each choice set contains various hypothetical alternatives with different attributes and levels, randomly combined by a rigorous method of DCE study design. Respondents are asked to choose one alternative that they prefer from each choice set. Finally, statistical analysis based on Random Utility Theory is used to determine the influences of attributes on respondent preference.

### Attributes and levels

We reviewed the clinical literature of treatments for patients with postmenopausal hormone receptor-positive, HER2-negative advanced breast cancer after failure of treatment with letrozole or anastrozole to develop a list of attributes for this study (Baselga et al. 2012; Bachelot et al. 2014; National Cancer Institute 2013; Yardley et al. 2013). Primarily, there were three attribute groups, including benefits [i.e (PFS), objective response rate, and clinical benefit rate], risks (i.e. stomatitis, anemia, hyperglycemia, fatigue, dyspnea, pneumonitis, rash, diarrhea), and costs. We used these attributes as a guide to conduct in-depth interviews with convenient sampled patients with breast cancer. These patients were asked about not only what attributes were important for them but also appropriate terms describing these attributes, assuming that they were in advanced state and standard treatments they had received fail. The interview data were transcribed verbatim after each interview. The data were saturated after interviewing six patients. Finally, we identified four attributes—PFS, anemia, pneumonitis, and monthly treatment cost—from only those attributes that were important for patients, as a result of the interview (Table 4). Level ranges were obtained from the same literature (Baselga et al. 2012; Bachelot et al. 2014; National Cancer Institute 2013; Yardley et al. 2013).

**Table 4:** Attributes and levels of treatments for patients with postmenopausal hormone receptor-positive, HER2-negative advanced breast cancer after failure of treatment with letrozole or anastrozole

Attributes	Levels
Progression free survival (PFS)	2, 8, 14 months
Anemia	0, 3, 6 %
Pneumonitis	0, 2, 4 %
Monthly cost	0, US$3030.3, US$6060.6^a^

^a^The exchange rate was approximately 33 Baht per US$1(Bank of Thailand 2014)

### Discrete choice experiment questionnaire design

**Fig. 1:**
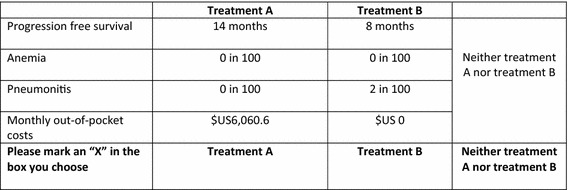
Choice set example

From all possible combinations of selected attributes and levels (3 × 3 × 3 × 3), it was not feasible to present them to an individual patient. An orthogonal and level balance design was used to randomly draw a subset of all combinations by using Ngene^®^ software (version 1.1.1). In this study, 36 choice sets were generated and divided into six blocks. A self-administered questionnaire was developed in Thai language. Each questionnaire contained six choice sets from each block. Therefore, we had six different questionnaires. Each choice set consisted of two alternatives describing hypothetical treatments and an opt-out alternative. Figure 1 shows an example of the choice sets. Another choice set was added to every questionnaire for a validity check. The validity check choice set contained a dominant alternative (highest efficacy, lowest side effect, and no cost), which must be chosen by patients who understood the questions. Questions on patients’ characteristics and experiences related to breast cancer treatments were included in the questionnaire. Three faculty members at Department of Pharmacy Administration, Faculty of Pharmaceutical Sciences, Prince of Songkla University were asked to check the content validity of the questionnaire before it was piloted with conveniently selected 10 breast cancer patients. No major problem was found.

### Data collection

This study conveniently sampled breast cancer patients, who visited the outpatient surgery department of a tertiary care hospital during May to September 2013 and aged 18 years old or above. Since the number of patients, who had postmenopausal hormone receptor-positive, HER2-negative advanced breast cancer treatments after failure of treatment with letrozole or anastrozole, were small, it was not possible to select only them in the study. Breast cancer patients at any stage were included. However, they must experience surgery, radiotherapy, chemotherapy, hormonal therapy, or other cancer treatments, and had been in the follow-up period for at least 3 months in order to ensure that they had some experiences with cancer treatments. Since the choice sets used in this study were unlabeled choices, there was no sample size calculation formula (de Bekker-Grob et al. 2010). This study followed the suggestion from Rose and Bliemer for continually collecting data until all criteria were found statistically significant (Rose and Bliemer 2013). The ethics committee of the hospital, where patients were recruited, approved the study proposal. We explained all study details to patients. If they agreed to participate to the study, they needed to sign an informed consent.

Before we interviewed the patients by using the designed questionnaires, we briefly described and ensured that they understood about advanced breast cancer, its treatments, and side effects. We asked patients to imagine if patient were in the situation that had already had the standard treatments for advanced breast cancer and the treatments failed, then they were asked to choose two treatment alternatives and an opt-out alternative in each choice set. An example of a completed choice set was provided. Each respondent received only one questionnaire, which contained a total of seven choice sets.

### Data analyses

Based on Random Utility Theory, patients’ responses for each choice set were observed and analyzed in DCE (Lancsar and Louviere 2008). The following utility, that a patient i assigned to an alternative j, U_ij_, was estimated:

$${\text{U}}_{{{\text{ij}}}} = \beta _{0} + \beta _{{\text{1}}} {\text{PFS}}_{{\text{j}}} + \beta _{{\text{2}}} {\text{Anemia}}_{{\text{j}}} + \beta _{{\text{3}}} {\text{Pneumonitis}} + \beta _{{\text{4}}} {\text{Cost}}_{{\text{j}}} + \varepsilon _{{{\text{ij}}}}$$

where β_0_ is the constant reflecting patients’ preference for having treatment relative to no treatment, β_1_, β_2_, β_3_ are the coefficients or the mean attribute weights of PFS, anemia, pneumonitis, and cost, respectively, ε_ij_ is error term. Multinomial logit model by using Nlogit^®^ version 4 was used to estimate the utility model. The value of each coefficient indicated the relative importance of each attribute, while the sign of the coefficient reflected whether the attribute had a positive or a negative effect on utility or preference, as compared with the base level. The level of statistical significance was set at 0.05.

Marginal WTP of the attributes were calculated by taking the ratio of the mean attribute coefficient to the mean coefficient of cost attribute. Each of them represented how much one was willing to pay for a 1-unit change in the attribute. Krinsky and Robb method was used to estimate 95 % confidence intervals of WTPs of the attributes (Krinsky and Robb 1986). Finally, we calculated WTP for both exemestane and exemestane plus everolimus by multiplying the marginal WTP for each particular treatment’s attribute and its level changes, which were obtained from literature (Baselga et al. 2012; Bachelot et al. 2014; National Cancer Institute 2013; Yardley et al. 2013).
